# Effect of wearing a brassiere during low‐dose chest CT for lung cancer screening

**DOI:** 10.1002/acm2.70679

**Published:** 2026-07-07

**Authors:** Michal Cech, Eva Kocova, Iva Milerska, Jiri Jandura, Martin Hyrsl, Martina Koziar Vasakova, Pavel Ryska

**Affiliations:** ^1^ Faculty of Medicine Charles University Hradec Kralove Czech Republic; ^2^ Department of Diagnostic Radiology University Hospital Hradec Kralove Hradec Kralove Czech Republic; ^3^ Department of Diagnostic Radiology, Faculty of Medicine in Hradec Kralove Charles University Hradec Kralove Czech Republic; ^4^ The Czech Institute of Informatics Robotics and Cybernetics Czech Technical University Prague Czech Republic; ^5^ Department of Respiratory Medicine Thomayer Hospital Prague Prague Czech Republic

**Keywords:** image quality, low‐dose CT (LDCT), radiation dose, screening program, size‐specific dose estimate (SSDE)

## Abstract

**Objective:**

To assess the impact of wearing a brassiere during low‐dose chest CT (LDCT) examination on image quality and radiation dose.

**Methods:**

A comparative study included 87 patients who underwent paired LDCT examinations at the Hospital from February 2022 to July 2024. Each patient underwent a standard LDCT (SLDCT) without a brassiere and a modified LDCT (MLDCT) with a brassiere. Objective image quality was assessed using the standard deviation (SD) in predefined lung regions. Three experienced radiologists compared the image quality subjectively. For comparison of radiation doses between the protocols, we used standard dose indicators including CTDIvol, DLP, and SSDE. Clinical data were collected between February 2022 and July 2024.

**Results:**

Objectively, no statistically significant differences in SD or radiation dose were observed between MLDCT and SLDCT protocols. Median centering differences between paired scans were minimal (−8.8 mm), ensuring consistent comparisons. Subjective image quality was rated as equivalent or better for MLDCT in most cases, with a high level of interobserver agreement (95.4%).

**Conclusion:**

Wearing a brassiere during LDCT examinations caused no worsening of image quality or increase in radiation dose. It facilitates easier patient centering. These findings suggest that the modified protocol could be safely implemented in routine clinical practice. Further research could extend these observations to other chest imaging protocols.

## 1 INTRODUCTION

The utilization of diagnostic imaging procedures for screening purposes has been steadily increasing.^1^ Among these, computed tomography (CT) stands out as a significant imaging modality, offering a comprehensive range of clinical insights into a patient's condition. In clinical scenarios where low‐dose CT (LDCT) protocols are appropriate, these methods provide a significant reduction in radiation exposure while maintaining acceptable image quality.^2^

The general recommendation for CT examinations is to remove any items or clothing containing metal components that are in the area being examined. In the case of chest CTs, this mainly concerns brassieres containing metal parts. In many institutions, it is standard practice that female patients are instructed to remove their brassieres before chest CT and to only wear a T‐shirt or hospital gown.^3^, ^4^

The requirement for female patients to undress fully during CT examinations may increase discomfort and anxiety; permitting them to retain their brassieres can reduce embarrassment and facilitate a more acceptable examination process.^5^, ^6^

Literature addressing the influence of brassieres on image quality and radiation dose in chest CT scans is scarce. To the authors' knowledge, studies on this topic have been published previously.^3^, ^7^

Existing literature indicates that the presence of personal metallic items or metallic components of clothing can influence radiation dose and image quality in CT examinations, particularly when automatic tube current modulation is used. Mulvey et al. conducted a pilot study evaluating the prevalence and impact of radiopaque personal items—such as zippers, brassiere underwires, and mobile phones—on CT dose and image noise in the context of ATCM. The authors found that small metallic objects (e.g., zippers or underwires) did not significantly affect dose or noise, whereas larger objects, particularly mobile phones, led to increased dose and noise when visible on the topogram within the planned scan range. These findings demonstrate that metallic objects present on the topogram can influence ATCM behavior and potentially compromise dose optimization.^7^

Seidenfuss et al. evaluated whether wearing a brassiere influences the performance of organ‐based tube current modulation (OBTCM) during chest CT. They demonstrated that a brassiere modifies breast positioning such that a larger proportion of breast and glandular tissue lies within the reduced‐dose projection angle, improving OBTCM effectiveness without compromising image quality. Metallic components did not generate diagnostically relevant artefacts. Their findings indicate that, under appropriate modulation strategies, the presence of a brassiere may support dose optimization.^3^

Our study extends the work of Mulvey et al. by evaluating a personal metallic item—specifically, a brassiere—as a distinct category of object.^7^ It also differs from Seidenfuss et al. by evaluating brassiere retention in a low‐dose lung screening CT protocol without OBTCM.^3^ The protocol used X‐CARE modulation (Siemens, Erlangen). In our study we used CAREDose 4D, a combination of z‐axis and angular modulation with online correction from the last 180° of the CT part of the scan rotation. To date, no study has evaluated the effect of wearing a brassiere in the context of low‑dose CT lung screening or low‑dose screening protocols in general.

The aim of our study was to verify whether it is possible to leave the brassiere on patients during LDCT examinations, considering both radiation exposure and, above all, image quality, which was assessed objectively and subjectively.

## 2 MATERIALS AND METHODS

### 2.1 Patient group

This comparative study analyzed female patients enrolled in the Pilot Program for Early Detection of Lung Cancer at the Hospital between February 2022 and July 2024, meeting strict inclusion criteria, and undergoing repeated LDCT scans. Each patient underwent two paired low‐dose chest CT scans: one using the standard low‐dose CT protocol (SLDCT) and the other using a modified low‐dose CT protocol (MLDCT). These scans were part of routine clinical care and were not associated with any additional procedures. Participating patients had at least one comparative scan, with intervals ranging from one month to two years.

This study was conducted in accordance with the Declaration of Helsinki, and approved by the Ethics Committee of University Hospital Hradec Kralove for studies involving humans. Informed consent was obtained from all subjects involved in this study.

Patient selection involved ensuring correct positioning by measuring the anteroposterior (AP) dimension and lateral (LAT) dimension at the T7 vertebral body's superior endplate.^8^, ^9^ The anterior‐posterior dimension is the thickness of the body part being scanned in the anterior‐posterior direction, for example from the surface of the stomach to the surface of the back, and the lateral dimension is the side‐to‐side (left to right) dimension of the body part being scanned.^10^ Along with the DICOM table height label, these measurements were considered.^9^ Accuracy of AP and LAT measurements was verified using an accredited phantom (ACR phantom ‐ Gammex 464—Sun Nuclear, Australia), with a mattress correction factor of +30 mm. Miscentering exceeding 30 mm between scans led to data exclusion, as it compromised image noise and radiation dose. Miscentering below the isocentre increased noise, while miscentering above isocentre elevated exposure and dose values.^8^, ^11^, ^12^

The final dataset comprised 174 paired examinations from 87 women who met all inclusion criteria (no motion artefacts, all CT scans were performed with the patient awake and fully conscious, and centering differences between scans were less than 30 mm). The average height was 1.63 ± 0.07 m, weight 74 ± 15.4 kg, and BMI 27.78 ± 5.38 kg/m^2^. Breast size was not assessed or considered in the selection process. The presence of brassiere reinforcement, whether metallic or non‐metallic, was noted on the CT scan. In total, 654 CT scans were reviewed, which represents the number of procedures, not unique patients.

The data were collected prospectively in a random, non‐selective manner, disregarding patient morphology.

### 2.2 Patient´s centering accuracy and challenges

Miscentering was determined in the axial slice located at the upper surface of the T7 vertebral body. The T7 vertebra is located at the center of the investigated area and the axial cross‐section is therefore suitable for patient centering evaluation or SSDE estimation. Miscentering can be calculated as one‐half of the patient's size in the AP dimension, from which we subtract the table height without the mattress correction as shown in equation 1.

(1) $$\mathrm{miscentering}=\frac{AP}{2}-\mathrm{TableHeight}+\mathrm{mattress}\;\mathrm{height}$$

**FIGURE 1:**
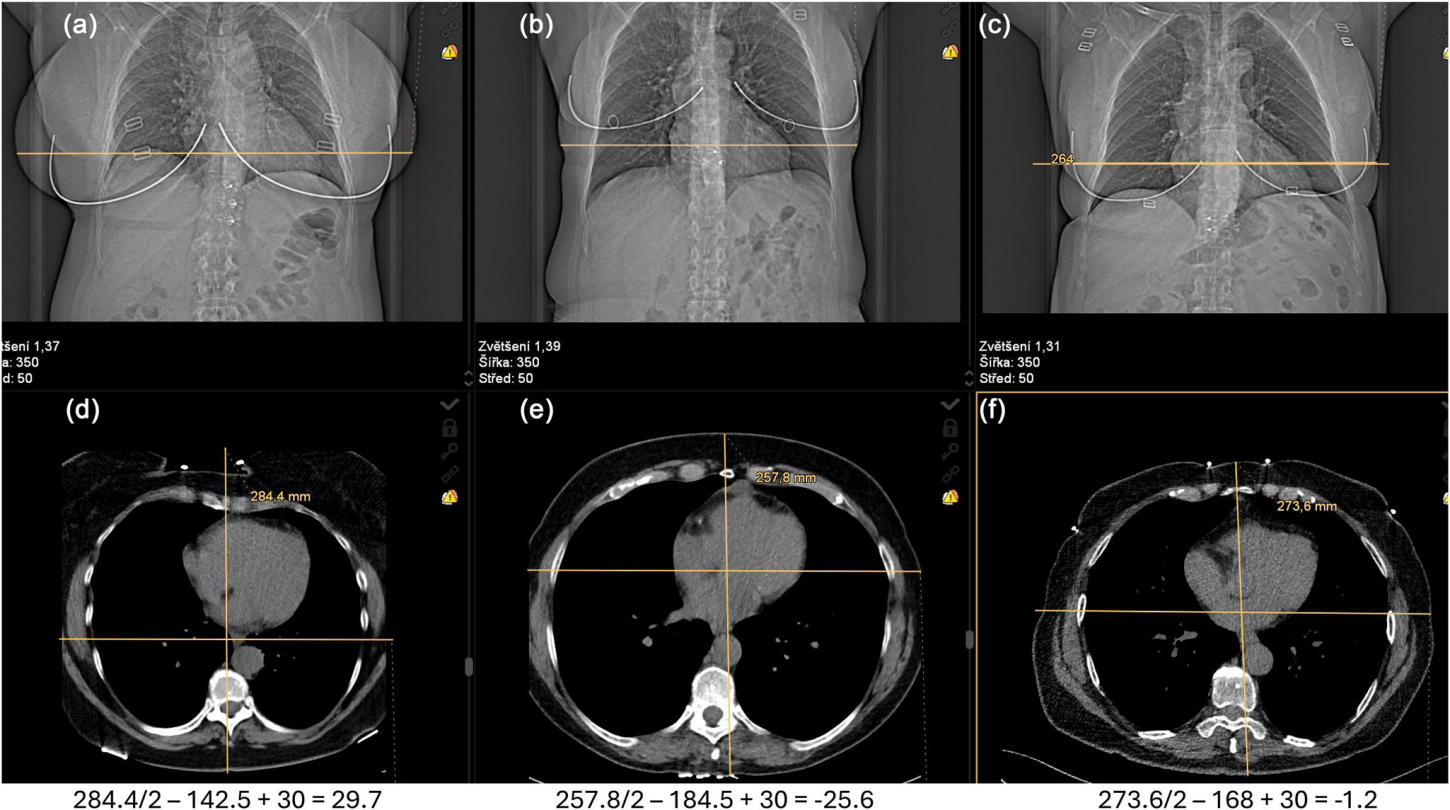
Illustrates the estimation of miscentering in relation to the gantry isocentre, picture (a) and (d) illustrate patient location above the isocentre, (b) and (e) illustrate patient location below the isocentre and c) and (f) illustrate patient location with minimal difference to isocentre.

The DICOM value of Table Height Attribute (0018,1130) of “0” corresponds to the table surface (without the mattress) being at isocenter, and positive values for “TableHeight” correspond to lowering the table from this position. In our study, a mattress with a height 30 mm was used. Miscentering differences between paired studies were less than 30 mm, because greater miscentering between studies can lead to increased radiation doses in positive miscentering and higher noise levels in negative miscentering.^13^, ^14^, ^15^ Miscentering values of average, median and SD were −10.05 mm and −7.2 mm; medians were −10.15 mm and ‐6.57 mm; SD values of miscentering 11.1 mm and 12.9 mm for SLDCT and MLDCT, respectively. This relationship is straightforward for AP topograms but inverted for PA topograms.^16^, ^17^, ^18^, ^19^ The same principle applies to patient´s centering differences in paired scans. Additionally, Figure 1 illustrates the estimation of patient miscentering in relation to the gantry isocentre.^8^ Figure 1 shows three examples of patient placement: 1a and 1d illustrate placement above the isocenter, as calculated from the equation 1, namely that the patient's dimension is 284.4 mm, the table height is 142.5 mm and the resulting error value from the isocenter is 29.7 mm.

Figure 1b,e illustrates placement below the isocenter, as calculated from the equation 1, namely that the patient's dimension is 257.8 mm, the table height is 184.5 mm and the resulting error value from the isocenter is −25.6 mm.

Figure 1c,f illustrates placement at isocentre, as calculated from the equation 1, namely that the patient's dimension is 273.6 mm, the table height is 168 mm and the resulting error value from the isocenter is −1.2 mm.

The correction for the mattress was included as a value of +30 mm, verified by measurements on the accredited phantom.

### 2.3 Standard and modified LDCT protocols

**FIGURE 2:**
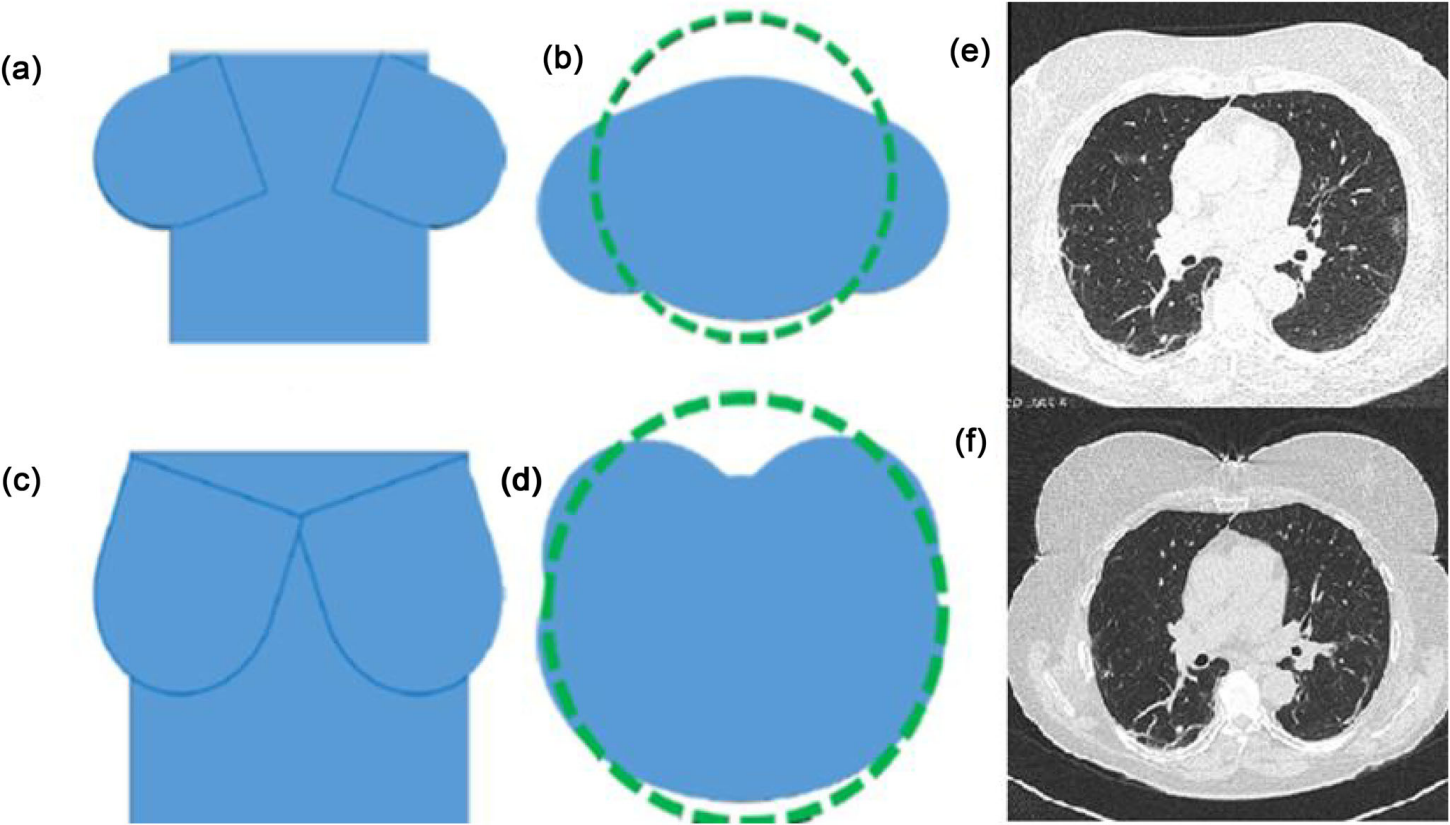
A schematic illustration depicts the differences in AP and axial views for scans without (a, b, e) and with (c, d, f) brassiere. Axial cross‐sections (e and f) further highlight these differences.^20^

The LDCT protocol parameters were established based on settings from the Siemens Somatom Definition AS+ (Erlangen, Germany), incorporating modifications for iterative reconstruction (IR) techniques. Key settings included an imaging voltage of 100 kV, a target image quality of 48 reference mAs, a pitch factor of 1, a rotation time of 0.33 s, and ATVS in mode AutokV set to “ON” and a modulation strength adjustable to 10. SLDCT scans were conducted with patients in a supine position, using helical mode with automatic exposure control (ATCM) and automated tube voltage selection (ATVS), covering the entire lung from apices to bases during full inspiration. Explanation, Figure 2 illustrates a cross‐section of the patient's body during SLDCT and MLDCT examinations.

In MLDCT, the protocol was identical to SLDCT except that patients retained their brassieres.

### 2.4 Subjective image quality

Subjective evaluation of image quality aimed at identifying changes and pathologies over time was conducted by three experienced radiologists. Each radiologist, with 16, 20 and 25 years of experience, assessed the overall image quality of paired examinations, comparing MLDCT (with brassiere) to SLDCT (without brassiere).^21^

They used a three‐level scale for assessment: +1 for better quality, 0 for similar quality, and −1 for worse quality.^22^ Fleiss kappa for analysis was calculated at 95% confidence level.

### 2.5 Objective image quality

Objective image quality was assessed using the SD value measurement in a 30×30 mm Region of Interest (ROI). SD is the standard deviation. ROIs were manually selected within the pulmonary lobes at identical slices for both SLDCT and MLDCT to ensure consistency. Measurements were taken at slices corresponding at T3, T5, T7, and T9, with both right (SDRP) and left (SDRL) lobes analyzed. Values were tabulated for comparison. The influence of texture and the degree of heterogeneity in the lungs were not taken into account in the measurements, because it depends on the exact respiratory phase, although all examinations in our study were performed in full inspiration. The examination was not performed with spirometric control, because it is not required for CT Screening of the lungs. Heterogeneity was minimized by selecting ROI in homogeneous lung regions and matching slices between paired scans. We did not address the possibility of heterogeneity in the study.

### 2.6 Radiation dose

Radiation dose indicators such as CTDIvol (mGy) and DLP (mGy·cm) were used, reported based on a 32 cm reference phantom. Size‐Specific Dose Estimates (SSDE) were calculated by adjusting CTDIvol based on the patient's effective diameter. SSDE was calculated using the effective diameter given in equation 2, where AP is anterior‐posterior thickness and LAT is the left‐to‐right dimension of the patient.^10^

(2) $$Eff.diameter=\sqrt{AP\;x\;LAT}$$

In published studies, the effective dose is used to monitor the radiation exposure of patients. The ED could be used to calculate DLP and a k‐factor of 0.014 mSv/mGy·cm for the thorax, adhering to standard practices.^23^, ^24^ In our study, we did not calculate ED for comparison between SLDCT and MLDCT.

### 2.7 Statistical analysis

All data, except for subjective image quality assessments, showed non‐normal distribution as determined by the Kolmogorov‐Smirnov test. Consequently, the non‐parametric Wilcoxon signed‐rank test for the SLDCT and MLDCT exams was employed for statistical analysis. Inter‐rater agreement for subjective image quality evaluations was assessed using Fleiss’ kappa statistic, with values ranging from 0 (agreement by chance) to 1 (perfect agreement).^25^

Uncertainty intervals for the main outcomes for SSDE, CTDIvol, SD in image were reported at the 95% confidence level.

## 3 RESULTS

### 3.1 Patient group

This study compared two similarly composed groups regarding BMI, height, weight, breast size, and positioning, with a median patient´s centering deviation of −8.8 mm, indicating that patients were slightly below the isocentre.^8^, ^26^ In our cohort, 69 patients wore metal‐containing brassieres and 18 wore non‐metallic ones. The analysis of CTDIvol, SSDE, DLP, objective and subjective image quality did not demonstrate a statistically significant difference between the groups (metal and non‑metal) at the 0.05 significance level. However, given the smaller size of the second group, minor differences may have remained undetected. In our dataset, ATVS selected a voltage of 100 kV in all cases.

### 3.2 Objective image quality

Data analysis from individual axial slices at T3, T5, T7 and T9 of the right and left lung lobe regions indicated no statistically significant difference in SD value between the two paired exams on similar slices. Figure 3a–d shows the distribution of SD values for SLDCT and MLDCT at four anatomical levels (T3, T5, T7, T9), separately for the right and left lung. Each anatomical level contains four boxplots (R‐SLDCT, R‐MLDCT, L‐SLDCT and L‐MLDCT). Statistically significant differences (*p*<0.05) are marked with an asterisk, except at the T3 level on the right side, which showed a marginally higher median value. Table 1 provides a textual summary of statistical results and p‐values for all anatomical level and left and right side. Figure 3 provides a detailed distribution of sample SD values for MLDCT and SLDCT. In Figure 3 on each box, the central mark indicates the median, and the bottom and top edges of the box indicate the 25^th^ and 75^th^ percentiles, respectively. The whiskers extend to the most extreme data points not considered outliers, and the outliers are plotted individually using the ‘+’ marker symbol. Objective image quality was reported at the 95% confidence level.

FIGURE 3Distribution of SD values (HU) at T3(3a), T5(3b), T7(3c) and T9(3d) for the right and left lung region, comparing SLDCT and MLDCT. Each anatomical level contains four boxplots (R‐SLDCT, R‐MLDCT, L‐SLDCT, L‐MLDCT). Asterisks indicate statistically significant differences (*p*<0.05).

**Figure:**
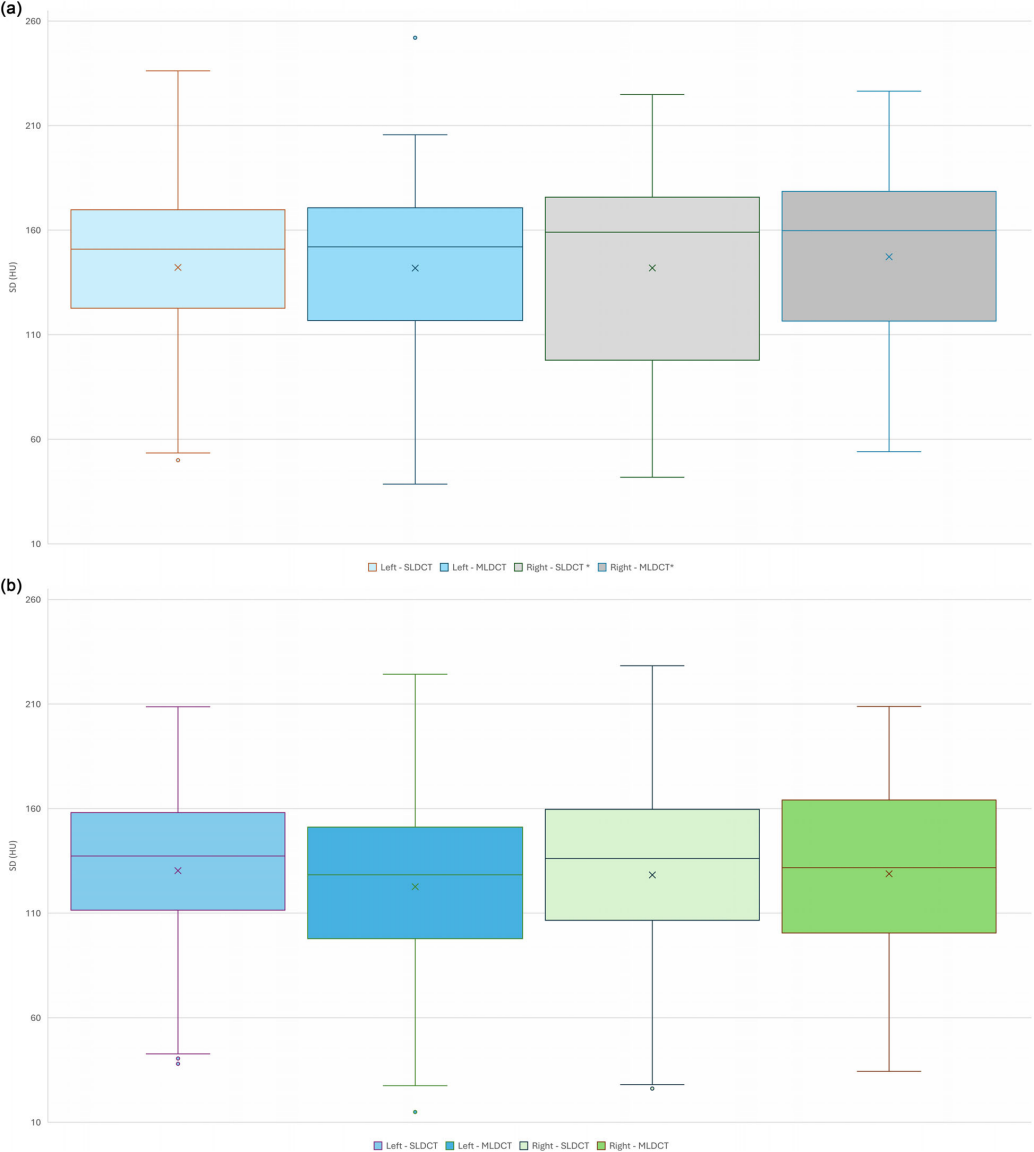

**Figure:**
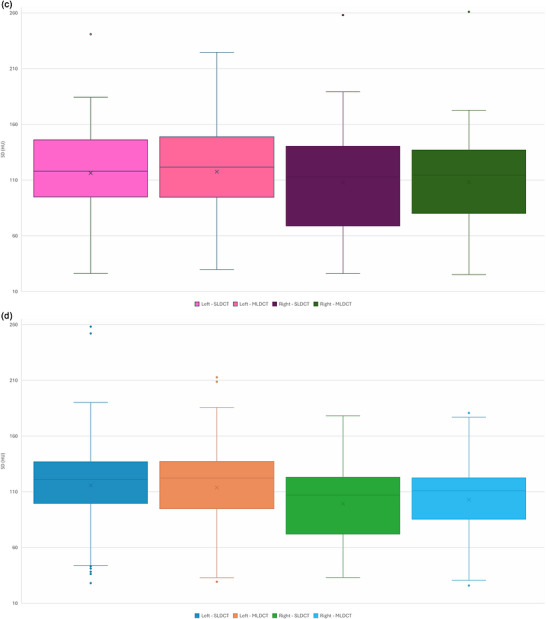

**TABLE 1:** Results from statistical analysis for SD values in slices and comparison between paired exams.

Slice position	Side location	*p*‐value	Results
**T3**	Left	0.6385	The difference between pairs of measurements is zero.
	**Right**	**0.0367**	**The difference between pairs of measurements is not zero**.
T5	Left	0.1041	The difference between pairs of measurements is zero.
	Right	0.4770	The difference between pairs of measurements is zero.
T7	Left	0.4640	The difference between pairs of measurements is zero.
	Right	0.9696	The difference between pairs of measurements is zero.
T9	Left	0.8889	The difference between pairs of measurements is zero.
	Right	0.1229	The difference between pairs of measurements is zero.

### 3.3 Subjective image quality

The evaluation results are summarized to indicate the number of evaluators agreeing on each score level (−1, 0, or +1). The Fleiss´ kappa was at the 95% confidence level. Fleiss' kappa calculation yielded a value of 0.27008, indicating a weak but non‐random agreement among the evaluators. Specifically: All three radiologists agreed in 38 out of 87 cases that the image quality was either the same or better for MLDCT.

At least two radiologists agreed in 83 out of 87 cases (95.4%) that the image quality was either the same or better for MLDCT. In four cases, no consensus was reached. There were three cases in which the subjective image quality was assessed by at least two radiologists as worse with MLDCT compared to SLDCT, in one case the radiologists did not agree on the assessment.

Table 2 displays the agreement rate in subjective image quality assessment of paired examinations by all three radiologists, including the observed variations.

**TABLE 2:** Display of the agreement rate in subjective image quality assessment of paired examinations by all three radiologists, including the observed variations.

Status of description	Number of cases
All three radiologists reported better IQ.	12
Two radiologists reported better IQ.	14
All three radiologists reported similar IQ.	26
Two radiologists reported similar IQ.	31
Two radiologists reported worse IQ.	3
All three radiologists reported different IQ (1 better, 1 similar and 1 worst).	1

### 3.4 Radiation dose

The evaluation of radiation dose indicators revealed no significant differences in SSDE and DLP between SLDCT and MLDCT exams. Our results showed *p*‐values of 0.15997 for SSDE, supporting the hypothesis of no significant difference between the protocols. SSDE and DLP values were reported at the 95% confidence level. CTDI_vol_ and DLP also showed no significant differences between SLDCT and MLDCT groups, with p‐values of 0.086533 and 0.21571 respectively, indicating no statistically significant differences. Average values, medians and SDs of SSDE and DLP for SLDCT and MLDCT in Table 3.

**TABLE 3:** Data of average values, median values and SD values of SSDE and DLP, for SLDCT and MLDCT protocol.

	SSDE			DLP		
	Average (mGy)	Median (mGy)	SD (mGy)	Average (mGy.cm)	Median (mGy.cm)	SD (mGy.cm)
Protocol SLDCT	2.52	2.46	0.645	71.21	67.5	24.26
Protocol MLDCT	2.45	2.38	0.625	69.68	65.5	25.99

For evaluating statistical significance or assessing the impact, it is not necessary to calculate specific ED values.

**FIGURE 4:**
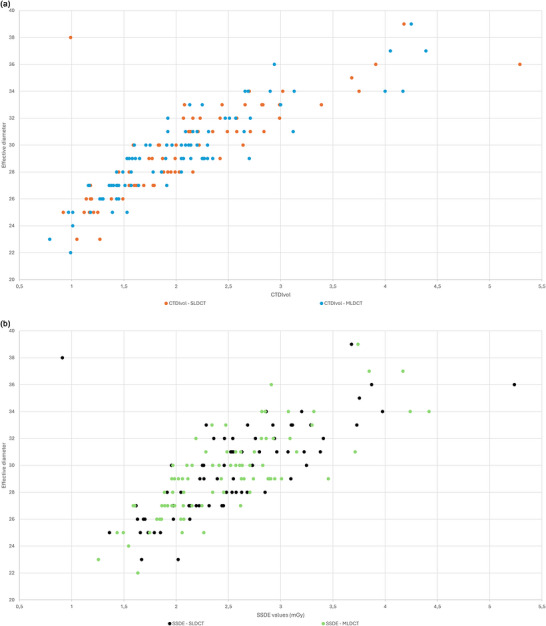
(a) Scatter plot to visually display the dependence of CTDIvol on patient size, represented by the effective diameter for SLDCT and MLDCT protocol. (b) Scatter plot to visually display the dependence of SSDE on patient size, represented by the effective diameter for SLDCT and MLDCT protocol. **Note**: The data suggest no statistically or clinically relevant differences were observed between scans with and without brassieres radiation dose levels, supporting their comparability.

Figure 4 shows a scatter plot showing the relationship between CTDIvol and SSDE values on patient size for SLDCT and MLDCT. The effective diameter was used to display patient size. The minimal differences in the cluster of points in the middle of the graph indicate that both CTDIvol and SSDE values can be used to assess the statistical difference between SLDCT and MLDCT.

## 4 DISCUSSION

### 4.1 Patient group

The analysis included 87 female patients, each of whom underwent at least two CT scans: one with and one without a brassiere. This resulted in 87 paired examinations consisting of high‐quality, well‐matched scans suitable for comparison and analysis. Image quality was evaluated subjectively by three consultant radiologists, while objective image quality was assessed by standard deviation (SD). The patient group was further subdivided into 69 patients wearing metal‐containing brassieres and 18 patients wearing non‐metallic brassieres. No statistically significant differences in either image quality or in dose indices were observed between these subgroups.

In our dataset, ATVS selected voltages only of 100 kV, which was probably caused by the higher values of strength modulation in ATCM (“10”). The CT device has higher operability in this setting using the current value.

All examinations were of sufficient quality to answer the clinical questions in accordance with standard diagnostic radiology practice. Using paired examinations minimized the influence of these variables on image quality, as both scans were acquired in the same patient within a short time interval.

### 4.2 Patient centering accuracy and challenges

Data required for SSDE estimation were collected manually, as the licence for the commercial system designed for data collection was not available in the hospital. Consequently, minor discrepancies between system‐derived values and manually measured values cannot be excluded. All paired examinations were included in the radiation dose comparison. Consequently, minor differences between system reported and manually verified values may occur; however, all dose values were retained in the dataset, and retrospective comparison confirmed that the extreme cases were consistent for both brassiere and non‐brassiere examinations.

To provide the most reliable results possible under these conditions, we decided not to include all potentially available values in our analysis that were out of range due to paired mis‐centering. Manually measured data were not validated with values obtained by commercial automatic software. Manual measurement values used to verify centering and obtain values for SSDE calculation included primarily the assessment of anteroposterior (AP) and lateral (LAT) dimension, table height and correction for mattress thickness.^8^, ^9^ Those steps were undertaken primarily to achieve sufficient accuracy of patient centering necessary for a relevant comparison of the paired scans.

### 4.3 Experimental protocol

The imaging protocol for the Pilot Program for Early Detection of Lung Cancer was thoroughly optimized so that it would meet legislative and clinical standards.^27^, ^28^ Those standards included primarily correct patient positioning, imaging voltage selection and appropriate training of the radiological staff. One of the key aspects of the protocol was the use of AP topograms instead of PA or LAT topograms, as it significantly reduces radiation exposure (by 10%–35%).^14^, ^15^, ^29^ When identical exposure parameters are used, an AP topogram can increase radiation exposure to the mammary gland by up to 70% compared with a PA topogram. In our MLDCT protocol, the AP topogram settings were decreased and modified from the manufacturer's default values 120 kV and 35 mA to 80 kV and 20 mA, which minimized breast dose while still providing adequate information for scan planning. This reduced the topogram DLP from 4–5 mGy.cm to 0.8–1 mGy.cm, and the adjustment resulted in an approximately 70% reduction in DLP, consistent with the feasibility and acceptability reported by Bohrer et al.^30^

Accurate centering improves the performance of ATCM regardless of topogram direction. In an AP topogram, certain anatomical structures (the spine, paraspinal ribs, and scapulae) are positioned closer to the detector than under otherwise identical conditions with a PA topogram, which may reduce geometric magnification. This results in a more accurate longitudinal dose profile for both longitudinal and angular modulation. When combined with CARE Dose4D online modulation, the overall radiation dose from the CT examination is reduced. The protocol further consisted of automated imaging voltage selection, a similar level of image quality and a precisely defined scanning range to minimize unnecessary radiation and to avoid incidental findings.

In the modified protocol, female patients were allowed to keep their brassieres on during scanning. The presence of a brassiere may slightly alter breast positioning, but does not affect global dosimetric quantities.

Seidenfuss et al.^3^ reported that glandular dose increases when breast tissue lies within the lateral projection of the X‐ray beam. In examinations where the brassiere remained in place, the breast was partially supported and displaced anteriorly, resulting in less glandular tissue located in the lateral beam path. This positional effect does not alter the water equivalent diameter and therefore does not conflict with the observation by Markovich et al.^31^ that the planar average dose (CTDIxsec) is independent of body ellipticity when cross‐sectional area is preserved. The observed dose differences in our dataset are small, and the anatomical redistribution of glandular tissue provides one possible explanation consistent with the published literature, without implying changes in global dosimetric quantities.

### 4.4 Objective image quality

Despite the importance of radiation dose, it is the image quality that is essential for detecting pathologies and subsequently for appropriate clinical management of the patient. Thus, we decided to evaluate the quality in this study both objectively and subjectively. For the objective part of the analysis, the standard deviation (SD) was used.

Clinically insignificant measurement inaccuracies occurred due to imperfect alignment of the images, as discussed above.^22^ Still, it is possible to conclude that wearing a brassiere during the examination did not negatively affect its diagnostic yield. Objective image quality and SD values may be further influenced by lung texture, patient inspiratory rate, alignment of axial slices used to measure SD, miscentering, reconstruction field of view (FOV) and accuracy of measurement ROI placement in the lung.

### 4.5 Subjective image quality

As subjective evaluation is often prioritized in clinical practice, the central focus of the study remains on the subjective part of the image quality. Each pair of the 87 cases was reviewed by three consultant radiologists with 16, 20 and 25 years of professional experience, respectively. We consider the target condition to be image quality similar to or better than MLDCT. Agreement between at least two radiologists regarding image quality being similar or better achieved in 83 cases. In three cases, two radiologists rated the image quality as worse, and in one case no consensus was reached. All four cases remained diagnostic. Those cases were reviewed afterwards. The study also indicated that keeping brassieres on during CT scanning slightly improved the overall image quality. We used a three‐point scale for the evaluation as it is sufficient to distinguish differences in image quality in a clear and uncomplicated way.^12^ However, image quality is always evaluated comprehensively. Therefore, aspects such as high and low contrast, image edges and noise are always part of a subjective assessment.^22^, ^32^ The use of paired examinations for each patient helps reduce the effects of these aspects. Subjective evaluation cannot be fully blinded because the presence or absence of a brassiere is clearly visible on reconstructed CT images. In this study, one of the radiologists was unaware of the study hypothesis and performed the evaluation independently.

Although higher tube voltage can reduce metal‑related artefacts,^33^ increasing kVp does not necessarily increase radiation dose because the required mAs can be substantially reduced. In the context of low‑dose screening protocols, however, the use of higher kVp remains undesirable, as the primary objective is to maintain the lowest feasible exposure while preserving diagnostically acceptable image quality.

### 4.6 Radiation dose

Patient‐specific Monte Carlo simulations are required for accurate effective dose estimations. However, for routine clinical comparison CTDIvol and DLP are often used instead.^10^, ^34^ To relate the radiation doses to the patient's constitution (e.g., BMI), we used Size‐Specific Dose Estimates (SSDE)to improve comparison of the radiation dose data by eliminating patient's variable factors such as BMI, height or weight. Addressing the common underestimation of CTDIvol values for small patients and the overestimation for the larger ones can be addressed by using SSDE values.^35^ Correction of the data by these methods allowed us to better compare intra‐group differences in radiation doses.^34^, ^36^

Although Organ‐Based Tube Current Modulation (OBTCM) can reduce overall AP exposure by 25%–50%, the doses in other regions (e.g., lungs) may be increased instead.^37^, ^38^, ^39^ OBTCM is rarely used in LDCT protocols due to its limited effectiveness. It should also be emphasized that patient centering and well‐optimized planning scans are essential for accurate dose profiles and consistent image quality.^17^, ^18^

## 5 CONCLUSIONS

As no statistically significant or clinically relevant differences were detected in the observed parameters, both standard and modified low‐dose CT protocols could provide sufficient image quality for diagnostic purposes. The modified protocol, moreover, allows female patients to keep their brassieres on while undergoing examination. Radiation dose indicators and radiation exposure levels in both protocols showed no statistically significant difference. These findings might support the clinical applicability of MLDCT in lung cancer screening programs.

## 6 CLINICAL RELEVANT STATEMENT

This study focused explicitly on comparing image quality and radiation dose parameters, emphasizing that no data regarding patients´ comfort or experience during examination were directly recorded. Therefore, it is only an assumption that allowing female patients to keep on their brassieres during examination might provide them with potential benefit from reducing stress, anxiety or embarrassment. Also, the acceptability of wearing a brassiere during CT examination should not be generalized, as the study analysed data specifically from The Pilot Program for Early Detection of Lung Cancer. Interchangeability of the protocols in different clinical scenarios (e.g., routine chest examinations, polytrauma) still needs to be supported by further evidence.

## AUTHOR CONTRIBUTIONS

**Michal Cech**: Writing—original draft; Visualization; Validation; Resources; Methodology; Formal analysis; Data curation; Conceptualization. **Eva Kocova**: Writing—review & editing; Visualization; Methodology; Formal analysis; Data curation; Conceptualization. **Iva Milerska**: Writing—review & editing; Visualization; Validation; Software. **Jiri Jandura**: Writing—review & editing; Visualization; Data curation. **Martin Hyrsl**: Writing—review & editing; Visualization; Data curation. **Martina Koziar Vasakova**: Writing—review & editing; Visualization; Supervision. **Pavel Ryska**: Writing—review & editing; Visualization; Supervision; Data curation.

## CONFLICT OF INTEREST STATEMENT

The authors declare no conflicts of interest.

## ETHICAL APPROVAL

This study was conducted in accordance with the Declaration of Helsinki, and approved by the Ethics Committee of University Hospital Hradec Kralove (reference number: 20211 P07, Date: November 8, 2021) for studies involving humans. Informed consent was obtained from all subjects involved in this study.

## Data Availability

Due to privacy restrictions, supporting data are not publicly available.
